# High-Performance and Simply-Synthesized Ladder-Like Structured Methacrylate Siloxane Hybrid Material for Flexible Hard Coating

**DOI:** 10.3390/polym10040449

**Published:** 2018-04-17

**Authors:** Yun Hyeok Kim, Gwang-Mun Choi, Jin Gyu Bae, Yong Ho Kim, Byeong-Soo Bae

**Affiliations:** Wearable Platform Materials Technology Center, Department of Materials Science and Engineering, Korea Advanced Institute of Science and Technology (KAIST), Daejeon 34141, Korea; yh930428@kaist.ac.kr (Y.H.K.); gm-choi@kaist.ac.kr (G.-M.C.); dkssyd444@kaist.ac.kr (J.G.B.)

**Keywords:** methacrylate siloxane hybrid material, sol–gel process, ladder-like siloxane structure, nano-indentation test, elastic recovery

## Abstract

A high performance ladder-like structured methacrylate siloxane hybrid material (LMSH) was fabricated via simple hydrolytic sol–gel reaction, followed by free-radical polymerization. A structurally ordered siloxane backbone, the ladder-like structure, which is an essential factor for high performance, could be achieved by a short period of sol–gel reaction in only 4 h. This results in superior optical (Transmittance > 90% at 550 nm), thermal (T_5 wt % decomposition_ > 400 ℃), mechanical properties(elastic recovery = 0.86, hardness = 0.6 GPa) compared to the random- and even commercialized cage-structured silsesquioxane, which also has ordered structure. It was investigated that the fabricated ladder-like structured MSH showed the highest overall density of organic/inorganic co-networks that are originated from highly ordered siloxane network, along with high conversion rate of polymerizable methacrylate groups. Our findings suggest a potential of the ladder-like structured MSH as a powerful alternative for the methacrylate polysilsesquioxane, which can be applied to thermally stable and flexible optical coatings, even with an easier and simpler preparation process.

## 1. Introduction

Polymer–siloxane hybrid (PSH) materials or polysilsesquioxanes, which are class II hybrids of co-networks of organic polymers and siloxane backbone, exhibit good thermo-mechanical, electrical, and optical properties due to the synergetic effect of the siloxane backbone and organic polymer portion [1,2]. In addition, the varying properties of PSHs can be easily tuned by changing the polymer parts, sol–gel reactions, and polymerization [3,4,5]. Notably, the selection of the functional polymer groups is crucial to adjusting the chemical durability, thermal resistance, and degradability [6,7,8]. Methacrylate groups have the merits of fast curing by UV radiation, excellent optical transparency, and mechanical toughness [9,10,11,12]. Based on these advantageous properties, methacrylate resins are commonly used in cosmetics, adhesives, substrates, and hard coatings [13,14].

Polysilsesquioxanes with structural regularity, such as polyhedral oligomeric silsesquioxane (POSS) and ladder-structured polysilsesquioxanes, have attracted much attention because of their superior thermal and mechanical properties, compared to the random-structured polysilsesquioxanes [15,16]. However, the polysilsesquioxanes have critical issues related to their synthesis, such as a low yield, complicated synthetic methodologies, and limited selection of functional groups. In particular, to obtain a functional POSS, an additional substitution reaction is usually needed between the Si-H containing POSS and an allyl containing functional monomer [17,18]. The ladder-structured PSHs have been studied by many groups mainly in Japan; however, inconvenient synthesis methodologies and low yields are still problems [19,20]. Recently, Hwang et al. reported the synthesis of various functional ladder-like structured polysilsesquioxanes (LPSQs) synthesized by the sol–gel reaction with a solvent over ten days and compared the thermal and mechanical properties of the LPSQs with POSS, which have carbazole and phenyl groups [21,22,23]. Hwang et al. also reported a hybrid ionogel electrolytes using methacrylate LPSQs for lithium ion batteries, and Zhang et al. applied the methacrylate-phenyl LPSQs into LED encapsulation [16,24,25]. Despite of the high potency of LPSQs as optical and electrical application, their application area is still limited and the complicate and time-consuming synthetic process are issues.

In this paper, we report a high performance ladder-like structure methacrylate siloxane hybrid material (LMSH) with simple fabrication, which takes only four hours without additional solvents. LMSH exhibits superior thermal resistance (T_5 wt %_ > 400 ℃) and elastic recovery (W_e_ > 86%) than random-structured siloxane hybrid material and commercial cage-structured polysilsesquioxane. The high elastic recovery after loading force loading is calculated from nano-indentation test and confirmed by in situ SEM image. The LMSH has highly ordered structure, along with high methacrylate conversion rate, which enables a high density of organic/inorganic co-networking, resulting in excellent thermo-mechanical properties. We propose that LMSH is appropriate for mass production due to its simple synthesis and that LMSH also has potential for a transparent, durable, and flexible coating according to the Musil’s study.

## 2. Materials and Methods

### 2.1. Materials

3-(trimethoxysilyl)propyl methacrylate (MPTMS) (ShinEtsu, Tokyo, Japan), 0.1 N sodium hydroxide aqueous solution, 0.1 N hydrochloric acid aqueous solution, 2,2-dimethoxy-2-phenylacetophenone (Sigma Aldrich, St. Louis, MO, USA) were purchased and used without further purification. Commercial methacrylate POSS (MA0735) was purchased from Hybrid Plastic (Hattiesburg, MS, USA).

### 2.2. Synthesis of Methacrylate Oligo-Siloxane (MO) Resins

The ladder-like structured methacrylate oligo-siloxane (LMO) resin was synthesized with a hydrolytic sol–gel reaction of MPTMS and water with a base catalyst. 50 g of MPTMS and 7.25 g of 0.1 N sodium hydroxide aqueous solution were mixed in a 250 mL two-neck flask by stirring with a magnetic stirrer. The mixture was hydrolyzed at 65 ℃ for 1 h, and then was condensed at 80 ℃ for 3 h under a nitrogen gas flow. The synthesized oligo-siloxane resin was stripped at 85 ℃ under a vacuum condition to remove residue materials like water and methanol. Finally, the stripped resin was filtered through a 0.45 μm sized Teflon filter. The random-structured methacrylate oligo-siloxane (RMO) resin was made by the same process for the LMO resin, except that the catalyst aqueous solution was switched from 0.1 N sodium hydroxide to 0.1 N hydrochloric acid solution. The final methacrylate oligo-siloxane resins were stored at room temperature in a bottle.

### 2.3. Fabrication of Methacrylate Siloxane Hybrid (MSH) Materials

The methacrylate siloxane hybrid materials were fabricated by a two-step curing process classified into a free-radical polymerization and a post-annealing step. The hybrid materials were cross-linked between the methacrylate groups of the oligo-siloxane molecules by a radiating UV lamp. The BDK was added as an UV initiator at a 2 wt % of the total mass of the methacrylate oligo-siloxane resins. Free radicals were generated by radiating UV in an argon-atmosphere glove box to prevent oxygen quenching. The resins were poured into a 1 mm thick stainless-steel mold, and the hybrid materials were UV cured for 10 min, and then the bulks were thermal treated at 150 ℃ for two hours for complete free-radical termination. The ladder-like, random-, and cage-structured methacrylate siloxane hybrid materials (LMSH, RMSH, and CMSH) were respectively fabricated from the LMO, RMO, and M-POSS resin.

### 2.4. Characterization

A Fourier transform infrared (FT-IR) spectroscope (JASCO, Oklahoma City, OK, USA; FT-IR 4600) equipped with a ZnSe attenuated total reflection (ATR) accessory was used to confirm the formation of the MO resins, fabrication for the MSHs, and conversion of the methacrylate groups. All the measurements were performed at a resolution of 4 cm^−1^ in the range of 600–4000 cm^−1^. ^29^Si NMR spectroscopy (Bruker, Billerica, MA, USA; 400 MHz) was used to study the degree of condensation and the structures of the MO resins. Samples were prepared in 20 wt % of the chloroform-d. The size of the MOs was measured by a particle size analyzer (Malvern Panalytical, Malvern, UK; Zetasizer nano zs). The viscosities of the MO resins at 25 ℃, correlating with condensation of the resins, were obtained with a viscometer (Brookfield, Toronto, Canada; DV-2+ pro). The average molecular weights of the MO resins were calculated from a GPC system (JASCO, Oklahoma City, OK, USA; LC-4000). The optical transmittance of the MSH bulks were obtained in the 300–800 nm wavelength range with a UV–vis spectrophotometer (Shimadzu, Kyoto Prefecture, Japan; SolidSpec-3700). The ladder-like and random-structures of the oligo-siloxane network were confirmed by thin-film X-ray diffractometer (Rigaku, Tokyo, Japan; D/MAX-RC). The thermal properties of the MSH bulks were obtained by thermogravimetric analysis (TA instrument, New Castle, DE, USA; TGA 2050), and all measurements were performed under a nitrogen gas flow at 5 ℃/min heating rate from 50 to 700 ℃. The refractive index as a function of wavelength was measured by ellipsometer (J.A. Wollam, Lincoln, NE, USA; Alpha-Se). The MSH film was spin-coated about 5 μm on Si substrate and angle of incidence was 70°. After the measurement, the refractive index was obtained by Cauchy transparent model. In order to investigate the mechanical properties of the MSH bulk samples, Berkovich nano-indentation tests (Nanomechanics, Inc., Oak Ridge, TN, USA; iNano™) were carried out at a constant strain rate of 0.2%/s up to the maximum load of 40 mN. Hardness and effective modulus values were obtained using CSM mode based on Oliver–Pharr method and elastic recovery was calculated using areas under the loading and unloading curves. In addition, to visually confirm the elastic recovery of the LMSH, in situ nano-indentation was performed by the Hysitron PI 87 nano-indentation system with a Berkovich tip in FE-SEM SU5000 (HITACHI, Tokyo, Japan).

## 3. Results and Discussion

### 3.1. Preparation of Methacrylate Oligo-Siloxane Resins

**Figure 1:**
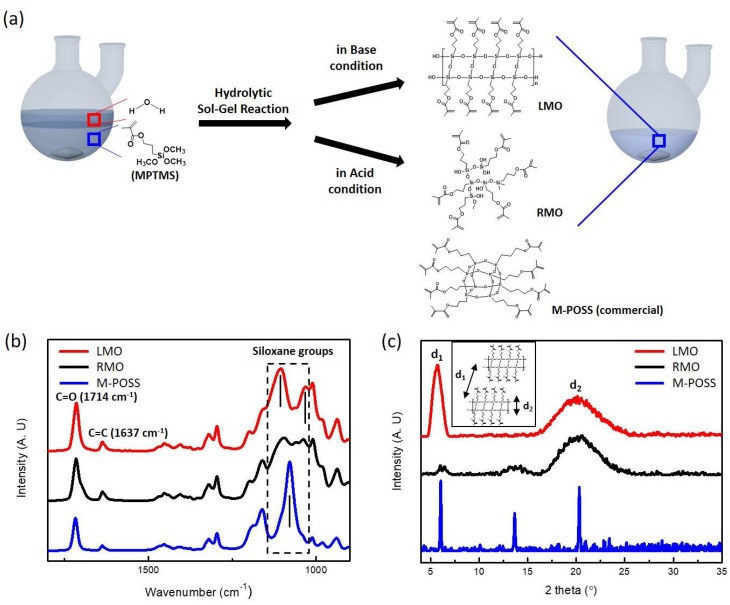
Molecular structures of the synthesized LMO, RMO, M-POSS resin. (**a**) Synthesis scheme of the methacrylate oligo-siloxanes via sol–gel hydrolysis and condensation; (**b**) FT-IR spectra of the MOs in the range of 900~1800 cm^−1^ (siloxane groups in the range of 1000~1150 cm^−1^); (**c**) thin film X-ray diffraction patterns of the methacrylate oligo-siloxane resins.

**Figure 2:**
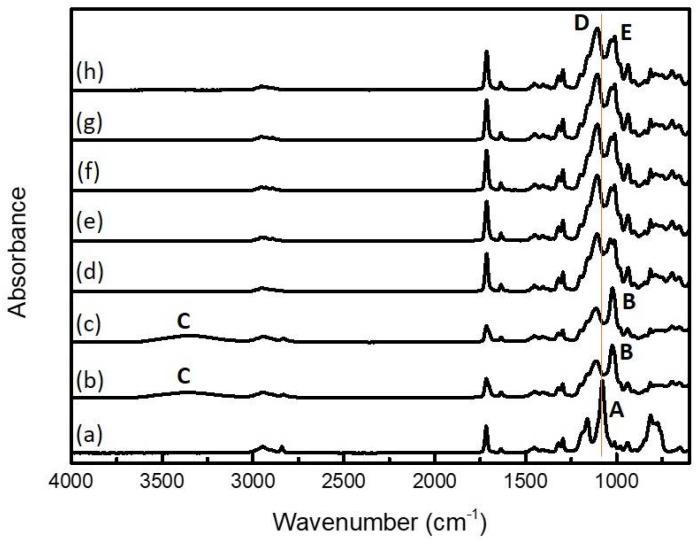
FT-IR spectra of LMO during sol–gel reaction: (**a**) MPTMS silane before sol–gel reaction; hydrolysis (**b**) 30 min and (**c**) 1 h; condensation (**d**) 1 h; (**e**) 2 h; (**f**) 3 h; (**g**) 6 h; and (**h**) 72 h.

Two methacrylate oligo-siloxane (MO) resins with different siloxane structures were synthesized via a mild hydrolytic sol–gel reaction of 3-(trimethoxysilyl)propyl methacrylate (MPTMS) with water as illustrated in Figure 1. The hydrolytic sol–gel reaction was simply processed for only 4 h without additional solvent. To promote the sol–gel reaction, hydrochloric acid or sodium hydroxide was added to water as an acid or base catalyst, and different structure of oligo-siloxane could be achieved by simply changing the kind of catalyst. According to the sol–gel chemistry, each catalyst shows different hydrolysis and condensation rate, resulted in different molecular structure of final siloxane network [26]. The expected molecular structures are illustrated as ladder-like structure MO (LMO, in base condition) and random-structured MO (RMO, in acidic condition) in Figure 1a. To comprehensively investigate the effect of the siloxane structures, a commercial methacrylate-POSS (M-POSS) was compared as a reference. The completion of sol–gel reactions of LMO/RMO were checked by FT-IR analysis over time during reaction (Figure 2). In the sol–gel process, methoxy groups (–OCH_3_) in MPTMS are hydrolyzed to hydroxyl groups (i.e., hydrolysis), then the hydroxyl groups are condensed with other methoxy or hydroxyl groups (i.e., alcohol condensation or water condensation, respectively). Compared to the MPTMS monomer (Figure 1a), a methoxysilane peak (Si–OCH_3_, **A**) was disappeared, and silanol groups (Si–OH, **B**) and hydroxyl groups (–OH, **C**) were appeared as result of hydrolysis of MPTMS (Figure 1b,c). The peak area of the silanol were saturated after 1 h of hydrolysis, then we stopped the reflux for hydrolysis and started sol–gel condensation by N_2_ purging. A set of two narrow siloxane peaks (Si–O–Si, **D** and **E**) at 1108 and 1133 cm^−1^ were formed as condensation process. As a time of condensation went by, the peak which corresponded to **D** increased compared to the one of **E**, and the LMO resin became optically clear and viscous with highly condensed siloxane networks. The ratio of **D** and **E** gradually increased with the progress of condensation reaction and saturated after 2 h from the start, and was even the same after 72 h (Figure 2d–h). So we extracted the LMO resin after 4 h of sol–gel reaction and used the LMO resin for a fabrication of methacrylate siloxane hybrid materials. The sol–gel reaction for the LMO resin is simple and fast, which is good for mass production and industrial applications.

### 3.2. Molecular Structure Analysis of the MO Resins

The molecular structures of LMO, RMO resins, and M-POSS were analyzed by FT-IR, ^29^Si NMR, XRD, and GPC, especially in terms of siloxane backbone structure. Figure S1a shows ^29^Si NMR spectra of LMO, RMO resins, and M-POSS. The ^29^Si NMR spectra confirm that all of the siloxane resins are consist of highly condensed Si species without any monomeric species, *T*^0^. In the denotation *T*^n^, “*T*” indicates the trimeric silicon species (MPTMS), and “n” stands for the number of siloxanes bound onto the Si atom. The degree of condensation (DOC) of the resins was calculated by the following equation [27].

$$\mathrm{Degree}\;\mathrm{of}\;\mathrm{condensation}\;(\mathrm{DOC})\;=\;\frac{T^{1}+2T^{2}+3T^{3}}{3\left(T^{1}+T^{2}+T^{3}\right)}\;\times 100$$

The calculated DOCs of the LMO, RMO, and M-POSS were 97.2, 88.7, and 98.2%, respectively. The ^29^Si NMR spectra of M-POSS shows only *T*^3^ species due to the purification process for the commercialization. In case of synthesized resins, LMO (base catalyst) shows higher DOC value than RMO (acid catalyst), because of catalyst and inductive effect. According to the sol–gel chemistry, acid catalyst promotes the protonation of the oxygen atom bound to the silicon atoms, while base catalyst promotes the deprotonated silanol before condensation step [26]. The charge of Si atoms become partial plus, as the condensation step progresses, it prefers the nucleophilic attack of the deprotonated silanol. Therefore, highly condensed siloxane networks, which are an essential condition for ordered siloxane structure, can be obtained when the base catalyst is used.

Figure 1b shows the FT-IR spectra of three different MO resins in the range of 900~1800 cm^−1^, and dotted rectangular box indicates the siloxane networks, which located in the range of 1000~1150 cm^−1^. Even though all of three resins have highly condensed siloxane networks, each resin has different siloxane structure, which can be confirmed by different peak position. The LMO shows a set of two narrow siloxane peaks at 1108 and 1133 cm^−1^, which corresponds to a horizontal and vertical stretching of the siloxane bonds, respectively. It should be noted that this set of siloxane peaks is usually observed in ladder-structured polysilsesquioxanes [20]. RMO, however, has broader multiple siloxane peaks, because randomly formed siloxane networks lead to less regularity of siloxane networks [28]. M-POSS has only a sharp siloxane peak at 1098 cm^-1^ due to its equivalence of every siloxane position [29].

As an additional structural analysis, the X-ray diffraction patterns of MOs were measured (Figure 1c). In the XRD patterns of the LMO, there is a set of two distinct diffraction peaks at 2θ = 5.8° and 20°, which is usually observed ladder-structured polysilsesquioxanes, which is in agreement with the result of the FT-IR analysis [30]. The peak at 5.8° is attributed to the intramolecular chain-to-chain distance, and the broad peak at 20° represents the average thickness in the ladder-structured polysilsesquioxanes [31]. On the other hand, the XRD patterns of the RMO shows only broad peaks, which confirms that the RMO has less regularity in siloxane structure like amorphous silicate glass [32]. Compared to the synthesized MOs, the M-POSS shows very sharp peaks at 6.1°, 13.7°, and 20.3°, because of the highest structural regularity that is originated from the purified silsesquioxane structure [33].

The properties of MO resins are summarized in Table S1, including GPC measurement for the molecular weight, viscosity measurement, and DLS measurement for the average molecular size (Figures S2 and S3).

### 3.3. Fabrication of the Methacrylate Siloxane Hybrid Materials (MSH)

**Figure 3:**
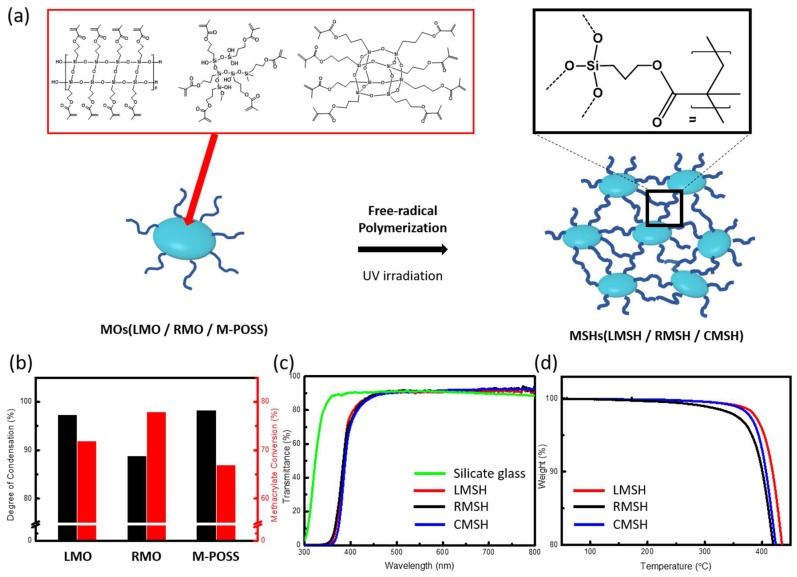
(**a**) Fabrication scheme of the methacrylate siloxane hybrid materials (LMSH, RMSH, and CMSH) with free-radical polymerization of methacrylate oligo-siloxane resins (LMO, RMO, and M-POSS); (**b**) Degree of condensation (black bar) and methacrylate conversion (red bar) of the resins; (**c**) Total transmittance of 1 mm bulks in the range of 300–800 nm and comparison of conventional silicate glass; (**d**) TGA spectra in a nitrogen atmosphere from 50 to 450 ℃.

As shown in Figure 3a, a ladder-like, random-, and cage-structured methacrylate siloxane hybrid materials (LMSH, RMSH, and CMSH) were fabricated by UV-initiated free-radical polymerization of methacrylate functions in the LMO, RMO, and M-POSS, respectively. The separated oligo-siloxane molecules were chemically bonded via the polymerization of methacrylate functional groups, result in MSHs, transparent rigid bulks.

The polymerization behavior of the three MOs were analyzed by FT-IR measurement using peak area decline of the carbon double bond groups (Figure S4). The conversion of methacrylate can be quantitatively calculated by comparing the polymerizable group (C=C, 1636 cm^−1^) to the non-polymerizable group (C=O, 1715 cm^−1^). The degree of methacrylate conversion was calculated with the following equation, based on the Beer–Lambert law [34].

$$\mathrm{Methacrylate}\;\mathrm{conversion}\;(\%)\;=\;(1-\;\frac{{\mathrm{Area}}_{C=O,\mathrm{MO}}}{{\mathrm{Area}}_{C=C,\mathrm{MO}}}*\;\frac{{\mathrm{Area}}_{C=C,\mathrm{MSH}}}{{\mathrm{Area}}_{C=O,\mathrm{MSH}}})\;\times 100$$

The calculated methacrylate conversion of the LMSH, RMSH, and CMSH were 71.8, 77.8, and 66.8%, respectively, meaning that all the MSHs were composed of highly cross-linked methacrylate networks as well as highly condensed siloxane networks. The methacrylate conversion of the LMO and M-POSS is lower than that of the RMSH due to their ordered structure. The ordered structure suffers steric hindrance and makes the oligo-siloxane difficult to twist during polymerization. Especially, the cage structure is the most rigid structure among three MOs, so the methacrylate conversion of the M-POSS has the lowest value.

### 3.4. Characterizations of the MSHs

The optical properties, thermal stability, and mechanical properties of the fabricated MSHs were measured for a durable optical applications. Figure 3c shows the transmittance of the MSHs in the range of 300–800 nm wavelength. All of the fabricated MSHs have excellent transparency over 90% in the visible range, and it is comparable to the conventional silicate glass. It was confirmed that the MSHs maintaining their high optical clarity even after the hybridization of the methacrylate and siloxane networks, which suggests that MSHs have no intra-/inter-phase separation that can degrade optical clarity of the hybrid materials.

Thermal decomposition of the methacrylate siloxane hybrid materials was examined by thermo-gravimetric analysis (TGA) with a 5 ℃ min^−1^ ramping rate under a nitrogen atmosphere, as shown in Figure 3d. All of the MSHs exhibited a superior thermal resistance, and the 5% weight decomposition temperature of the LMSH, RMSH, and CMSH was 404, 385, and 394 ℃, respectively. The high density of the siloxane and organic (i.e., methacrylate) co-networks contribute to the heat resistance of the organic methacrylate and methyl groups from the siloxane backbone during thermal pyrolysis. Both of the ordered siloxane hybrid materials (LMSH and CMSH) exhibit a higher thermal resistance than that of the RMSH due to the higher regularity of their structure and the higher degree of condensation of siloxane network. In particular, high DOC of siloxane network and high cross-linking of methacrylate of LMSH leads to the highest thermal stability among the MSHs. Despite the highest structural regularity and DOC of M-POSS, CMSH shows lower thermal stability than LMSH due to its lower conversion rate of methacrylate. However, since the repeating siloxane unit is equal, the ratio of organic to inorganic groups is the same, and residue weight of the three MSHs after 700 ℃ is the same (Figure S5).

The refractive index (RI) is a good indicator to compare the density of organic–inorganic co-networks [35]. The RI values of the MSHs at 633 nm, measured by prism coupler, are listed in Table 1. Figure S6 shows the refractive index of LMSH, RMSH, and CMSH as a function of wavelength, indicating that the LMSH has the highest RI value because of the high DOC and methacrylate conversion rate. It shows a consistence tendency with as in the case of thermal stability. Despite the DOC of the M-POSS is the highest, the CMSH has the lowest RI value, which originated from a large amount of free volume in the polyhedral siloxane of the M-POSS [36]. We additionally calculated the Abbe number, which used to classify glass and other optical materials in terms of their chromaticity, using RI values at 589.3, 486.1, and 656.3 nm. The Abbe numbers of LMSH, RMSH, and CMSH were 62.7, 56.2, and 71.4, respectively. The MSH shows a comparable Abbe number compared to fused silica (RI = 1.458, Abbe number = 67.7), which represents the suitability of optical coating materials.

**Figure 4:**
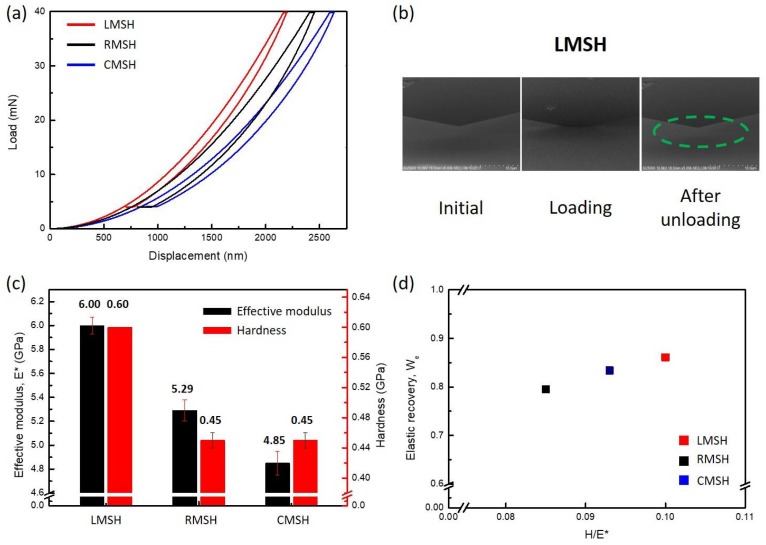
Mechanical properties of LMSH, RMSH, and CMSH with nano-indentation test. (**a**) Load versus indenter displacement for the methacrylate siloxane hybrid materials; (**b**) SEM images of the LMSH during nano-indentation test. The green dashed line is indicated the loading area, the impression is almost recovered after nano-indentation test (Video S1); (**c**) a plot of effective modulus and hardness data, in situ calculated during nano-indentation test; (**d**) a plot of the elastic recovery versus hardness-to-effective modulus ratio. The elastic recovery is derived by integrating the graph.

The mechanical properties of the MSHs, such as hardness, effective modulus, and elastic recovery, were obtained by nano-indentation measurements using Berkovich tip based on the Oliver–Pharr method [37]. The load-displacement curves for the MSHs are presented in Figure 4a, which shows that the LMSH exhibits the lowest hysteresis in the curve, which suggests the elastic absorption of the external force. Figure 4b shows in situ SEM images captured during the nano-indentation test of the LMSH (Video S1). It suggests that LMSH shows elastic deformation throughout the entire measurement range, so that the impression is fully recovered after unloading, along with the lowest hysteresis in the load–displacement curve (Figure 4a). Despite of the high degree of condensation and ordered polyhedral structure of the M-POSS, the CMSH is the largest deformation under the same load, because the free volume in the CMSH absorbs and relaxes the force by deforming its structure.

The effective modulus and hardness were obtained with respect to the indentation depth using the continuous stiffness measurement (CSM) technique (Figure S7), and the average values are shown in Figure 4c. The effective modulus/hardness of the LMSH reaches up to 6.00/0.6 GPa; while the value for RMSH are 5.29/0.45 GPa and for CMSH are 4.85/0.45 GPa. This result suggests that high effective modulus and hardness can be achieved by ladder-like siloxane backbone structure along with the high density of co-networks (See the supporting information for the raw values of multiple nano-indentation measurement, Tables S2–S4). Our LMSH exhibits an excellent mechanical performance compared with those of the methacrylate-phenyl ladder-like polysilsesquioxanes, fabricated by Hwang’s group; for example, values of effective modulus = 7.5 GPa, and hardness = 0.47 GPa were achieved [31].

Figure 4d shows a plot of the elastic recovery (W_es_) versus hardness over effective modulus ratio (H/E*). The elastic recovery was calculated by integrating the hysteresis in the load–displacement curves. The two regular-structured MSHs (LMSH and CMSH) have an excellent elastic recovery over 83%, compared to the 79.5% elastic recovery of the RMSH. Musil et al. suggested key parameters in the design of a flexible hard coating resistant to cracking [38]. In the study by Musil et al., the materials for flexible hard coating should have a high value for H/E* ≥ 0.1 and W_e_ ≥ 0.6. The RMSH and CMSH exhibit a high elastic recovery with W_e_ = 0.79 and 0.83; however, the H/E* ratio (0.085 and 0.093) is slightly lower than 0.1, which is insufficient for the flexible hard coating. Notably, the H/E* of the methacrylate-phenyl ladder-like polysilsesquioxane from Hwang’s group is 0.063, our LMSH is more appropriate for flexible hard coating according to Musil’s study, despite simple and fast synthetic sol–gel reaction. The LMSH has outstanding values which are H/E* = 0.1 and W_e_ = 0.86; thus, the LMSH is appropriate for use in flexible coatings. The high elastic recovery is even comparable with a commercial ceramic hard coating (Al-Cu-N film, W_e_ = 0.74), which was achieved by organic–inorganic hybridization without phase separation of organic–inorganic phase, along with high density of the organic–inorganic co-network [39].

**Figure 5:**
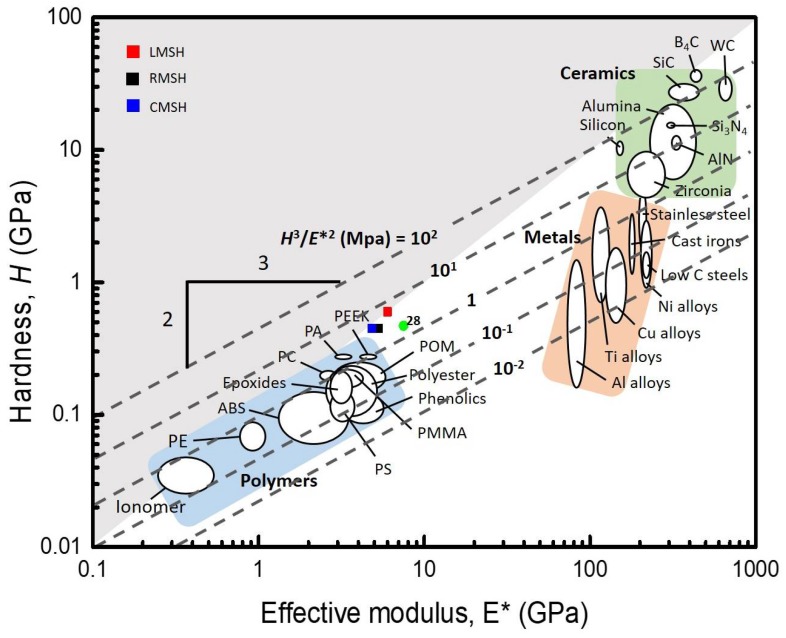
An Ashby map of hardness versus effective modulus for wear resistance. The LMSH only exists in the upper shaded area (H/E* ≥ 1). Values for other synthetic methacrylate-phenyl ladder-like polysilsesquioxanes are given in [31].

We illustrate the Ashby plot of the hardness versus the effective modulus to visualize the wear resistance of MSHs, compared with conventional polymers, metals, and ceramics (Figure 5) [40]. The shaded area indicates the upper value of H/E* ≥ 0.1, exhibiting a high hardness and low modulus, and the diagonal dashed line means the criteria of H^3^/E*^2^ which is related to the resistance to the plastic deformation during contact events [41]. LMSH is only located in the shaded area that indicates a polymer-like low effective modulus, which makes the coating be flexible like common polymers, as well as with a ceramic-like high hardness. It is a very unique value because common polymers, metals, and ceramics are located in the inferior white area. The LMSH also exhibits the highest H^3^/E*^2^ value, compared to conventional flexible polymers, and it is even comparable with ceramics. The combination of the polymer-like low E*, ceramic-like high H/E* and H^3^/E*^2^ values makes the LMSH a powerful candidate for optically transparent, flexible, wear resistant, and hard coatings. The optical, thermal, mechanical properties of MSHs are summarized in Table 1.

Water repellency is generally related to dirt retention, self-cleanability, and longer life expectancy of the coating itself. As an indicator of water repellency, water contact angles of MSHs were measured. In the case of conventional silicate glass, its surface is hydrophilic with high wettability (contact angle = 16.17°), so the surface of silicate glass can be easily contaminated from fingerprint or dust. The contact angle of the LMSH, RMSH, and CMSH were 81.82°, 86.29°, and 80.83°, respectively (Figure S8). The MSHs show higher water repellency than silicate glass without any surface treatment, which can cause degradation of mechanical properties and scratch-resistance, exhibiting the suitability of MSHs as optically clear hard coating materials.

## 4. Conclusions

In summary, we report a novel ladder-like structured methacrylate siloxane hybrid material (LMSH) with high optical transparency (90.6% at 550 nm), high thermal stability (T_5 wt % decomposition_ > 400 ℃), and robust mechanical properties (polymer-like low modulus, ceramic-like high H/E* and H^3^/E*^2^) that can be fabricated by simple sol–gel reaction and free-radical polymerization. The superior thermal resistance and mechanical properties of the LMSH originate from its molecular level hybridization, regularity of the siloxane structure, and the highest density of the siloxane-methacrylate co-networks compared to the random- and cage-structured MSHs. These results suggest that the properties of the siloxane hybrid materials can be controlled by modifying the siloxane backbone structure of the oligo-siloxane at a molecular level. The simple fabrication process and superior properties of LMSH suggest that is fit for application in optically transparent flexible hard coatings.

## Figures and Tables

**Table 1:** Values related to optical, thermal, and mechanical properties of methacrylate siloxane hybrid materials.

Sample	Methacrylate Conversion	Refractive Index at 633 nm	Transmittance at 550 nm	5 wt % Decomposition Temperature, T_5 wt %_	Hardness, H	Effective Modulus, E*	H/E*	Elastic Recovery, W_e_
	%		%	°C	GPa	GPa		%
LMSH	71.8	1.5043	90.6	403.77	0.6	6	0.1	86.1
RMSH	77.8	1.5032	91.3	384.76	0.45	5.29	0.085	79.5
CMSH	66.8	1.4988	91.1	393.83	0.45	4.85	0.093	83.4

## Supplementary Materials

The following are available online at http://www.mdpi.com/2073-4360/10/4/449/s1, Figure S1: (a) ^29^Si-NMR spectra and (b) Chemical Shift of Si depending on the bond state; Figure S2: (a) Viscosity of the LMO and RMO resin at 25 ℃. (b) GPC spectra of the LMO, RMO, and M-POSS resin; Figure S3: Dynamic light scattering spectra of the LMO, RMO, and mPOSS resins for the average molecular size; Figure S4: Methacrylate conversion of the MOs. The degree of methacrylate conversion is calculated by using the ratio of peak area of unreactive carbonyl groups (C=O) and reactive carbon double bond groups (C=C) in the FT-IR spectra, measured (a) before and (b) after the free-radical polymerization and post-annealing process; Figure S5: TGA spectra in a nitrogen atmosphere from 400 to 700 ℃; Figure S6: Cauchy-type dispersion curve (Refractive index curve) of LMSH, RMSH, and CMSH thin film measured by ellipsometer; Figure S7: In situ hardness and effective modulus values of the LMSH, RMSH, and CMSH; Figure S8: Water contact angles of (a) silicate glass, (b) LMSH, (c) RMSH, and (d) CMSH; Table S1: Values related to the molecular structures of three methacrylate oligo-siloxanes; Table S2: Nano-indentation results for the LMSH; Table S3: Nano-indentation results for the RMSH; Table S4: Nano-indentation results for the CMSH; Video S1: SEM image during nano-indentation test of LMSH (Plays at twice faster than original speed).
