# Supplementary material for: High-Performance and Simply-Synthesized Ladder-Like Structured Methacrylate Siloxane Hybrid Material for Flexible Hard Coating
Source: Polymers (Basel). 2018 Apr 17;10(4):449. doi: 10.3390/polym10040449 (PMC6415222; doi:10.3390/polym10040449)
Supplement: Supplementary file 1 [file polymers-10-00449-s001.zip › polymers-289087-suppl for proof.docx]

Article

High-Performance and Simply-Synthesized Ladder-Like Structured Methacrylate Siloxane Hybrid Material for Flexible Hard Coating

Yun Hyeok Kim, Gwang-Mun Choi, Jin Gyu Bae, Yong Ho Kim *, and Byeong-Soo Bae *

Wearable Platform Materials Technology Center, Department of Materials Science and Engineering,
Korea Advanced Institute of Science and Technology (KAIST), Daejeon 34141, Korea;
yh930428@kaist.ac.kr (Y.H.K.); gm-choi@kaist.ac.kr (G.-M.C.); dkssyd444@kaist.ac.kr (J.G.B.)

***** Correspondence: suktaiji@kaist.ac.kr (Y.H.K.); bsbae@kaist.ac.kr (B.-S.B.); Tel.: +82-42-350-5119 (B.-S.B.)

Other Supplementary Materials for this manuscript includes the following:

• **Video S1.** SEM image during nano-indentation test of LMSH (Plays 2 times faster than original speed)


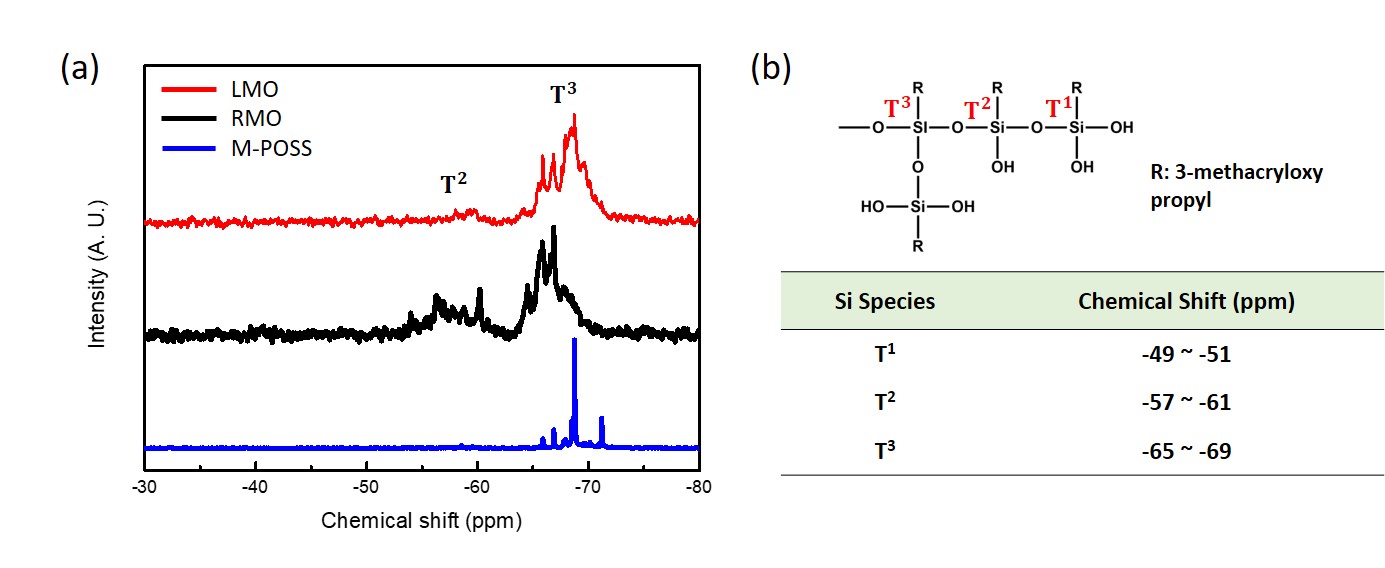


**Figure S1.** (a) ^29^Si-NMR spectra and (b) Chemical Shift of Si depending on the bond state.


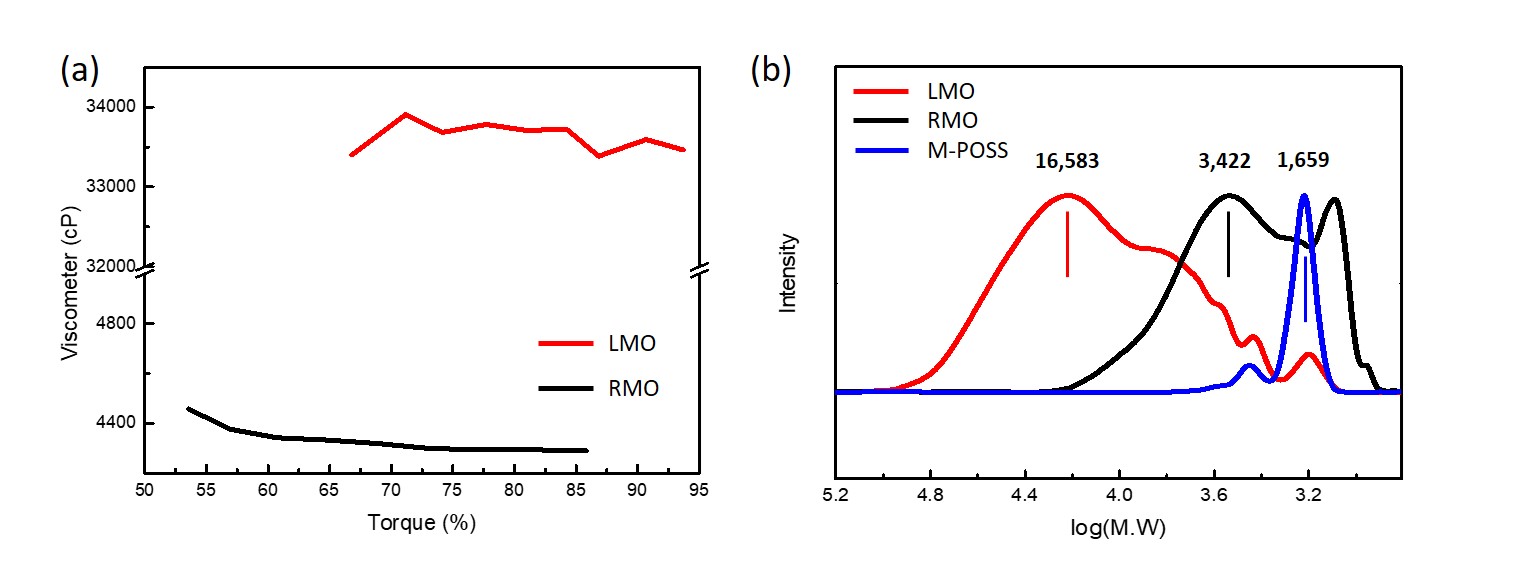


**Figure S2.** (a) Viscosity of the LMO and RMO resin at 25$℃$. (b) GPC spectra of the LMO, RMO, and M-POSS resin.


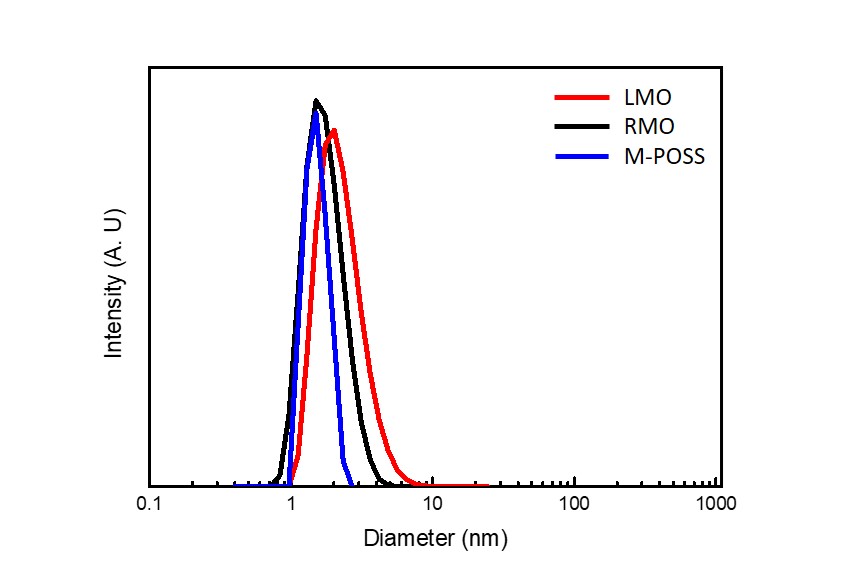


**Figure S3.** Dynamic light scattering spectra of the LMO, RMO, and mPOSS resins for the average molecular size.


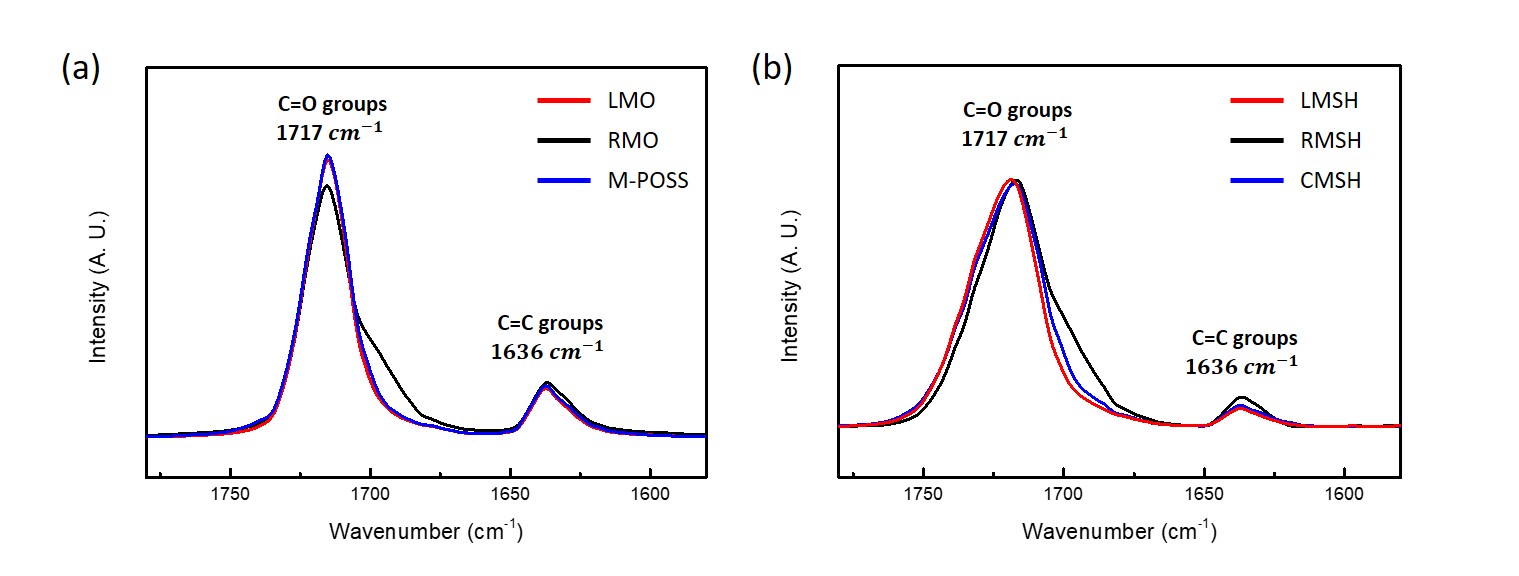


**Methacrylate Conversion(%)** = (1 - $\frac{\mathrm{Area}_{C=O, resin}}{\mathrm{Area}_{C=C, resin}} * \frac{\mathrm{Area}_{C=C, bulk}}{\mathrm{Area}_{C=O, bulk}}$) * 100

**Figure S4.** Methacrylate conversion of the MOs. The degree of methacrylate conversion is calculated by using the ratio of peak area of unreactive carbonyl groups (C=O) and reactive carbon double bond groups (C=C) in the FT-IR spectra, measured (a) before and (b) after the free-radical polymerization and post-annealing process.


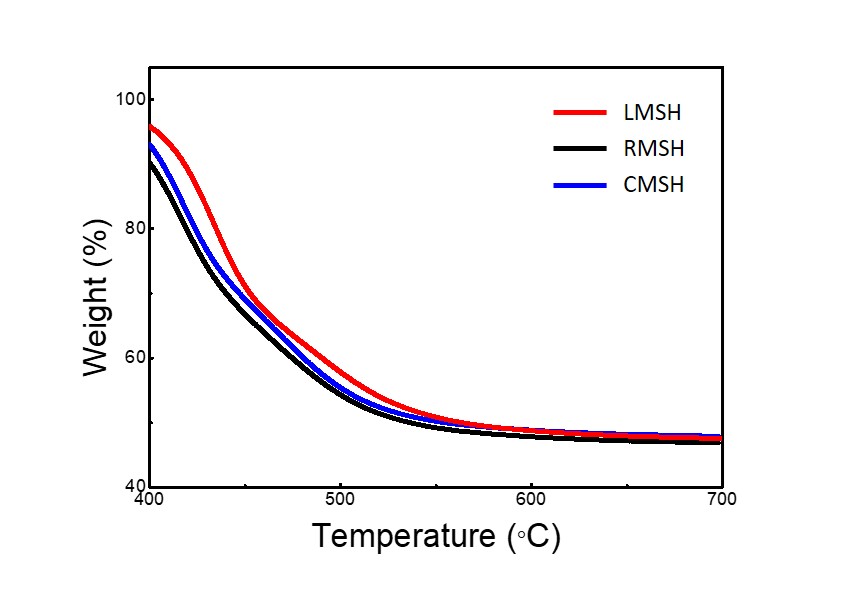


**Figure S5.** TGA spectra in a nitrogen atmosphere from 400 to 700 $℃$.


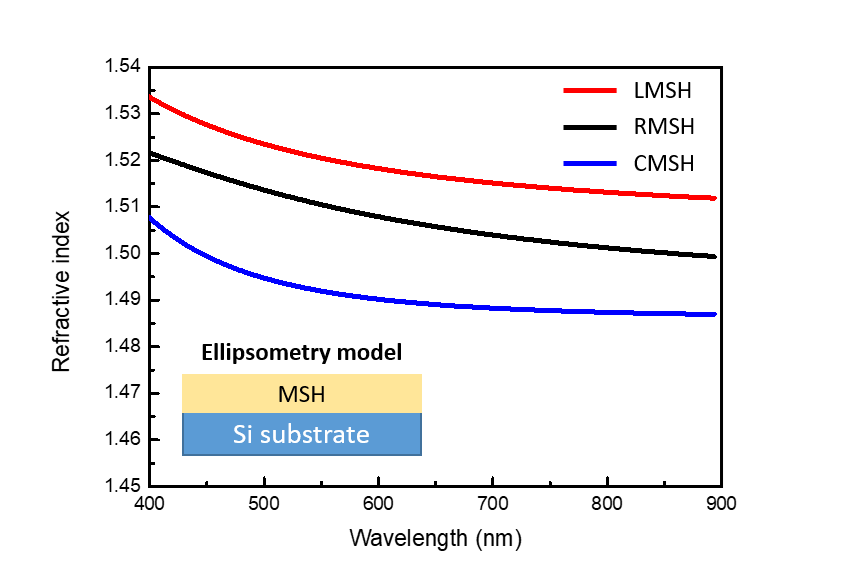


**Figure S6.** Cauchy-type dispersion curve (Refractive index curve) of LMSH, RMSH, and CMSH thin film measured by ellipsometer.


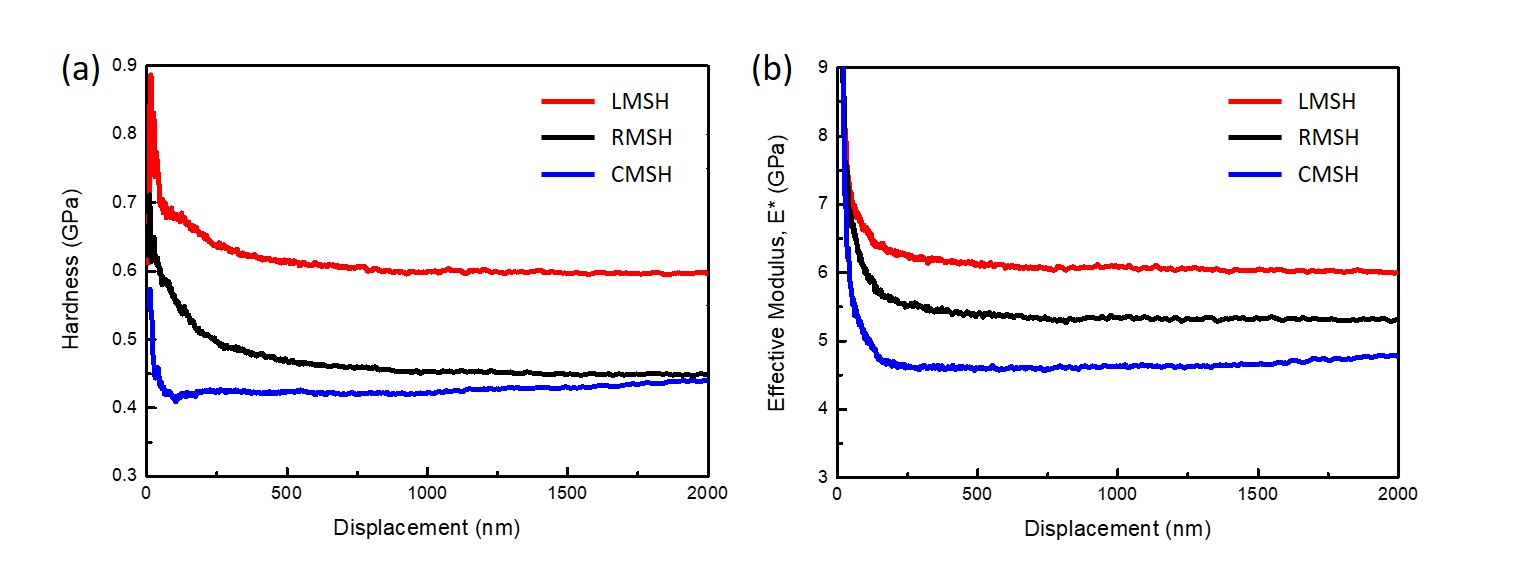


**Figure S7.** *In situ* hardness and effective modulus values of the LMSH, RMSH, and CMSH.


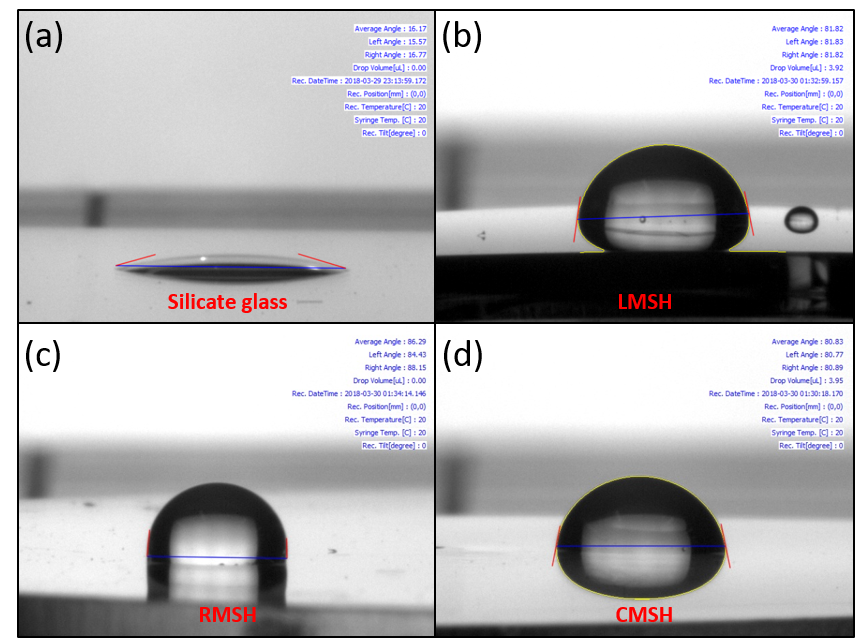


**Figure S8.** Water contact angles of (a) silicate glass, (b) LMSH, (c) RMSH, and (d) CMSH.

**Table S1.** Values related to the molecular structures of three methacrylate oligo-siloxanes.


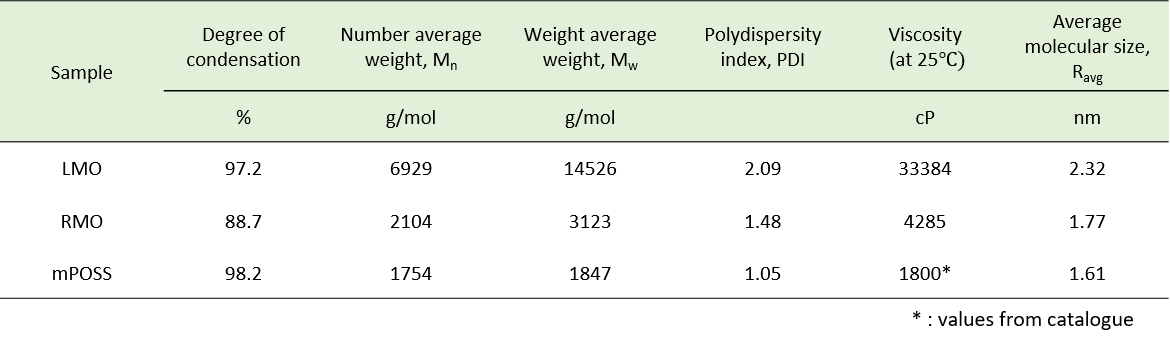


**Table S2.** Nano-indentation results for the LMSH.


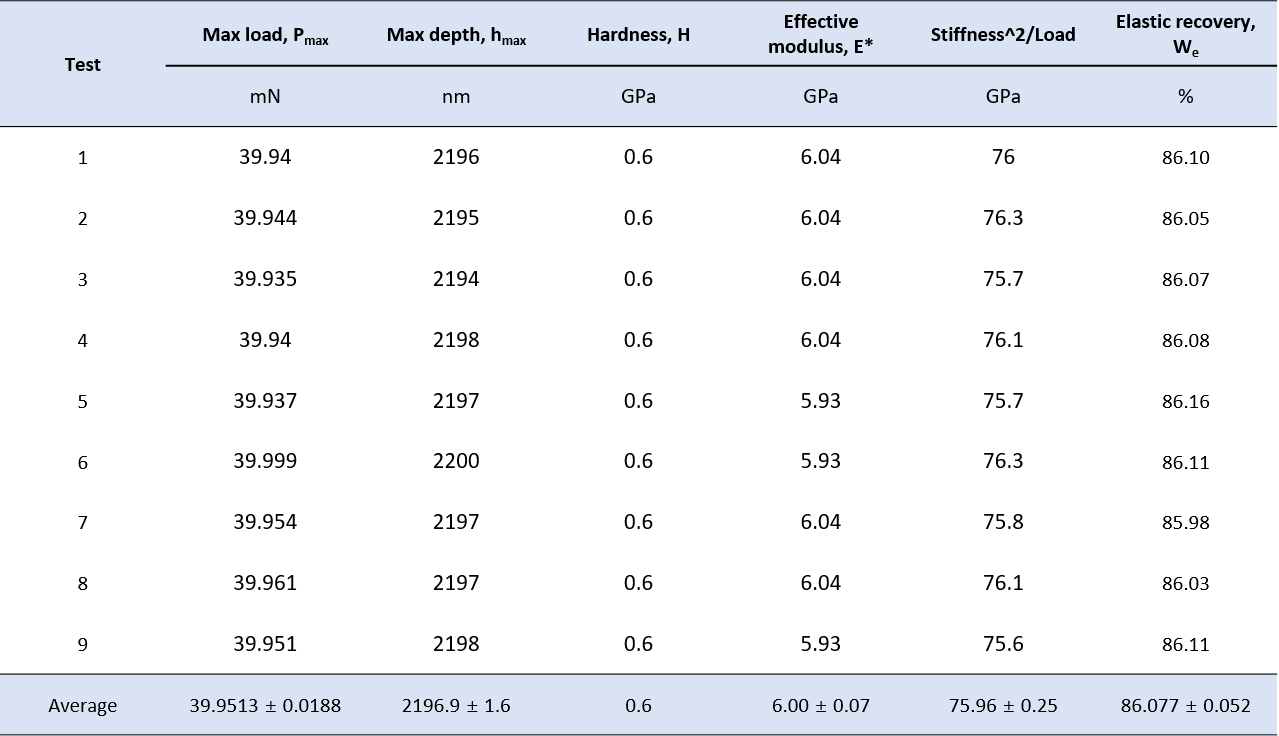


**Table S3.** Nano-indentation results for the RMSH.


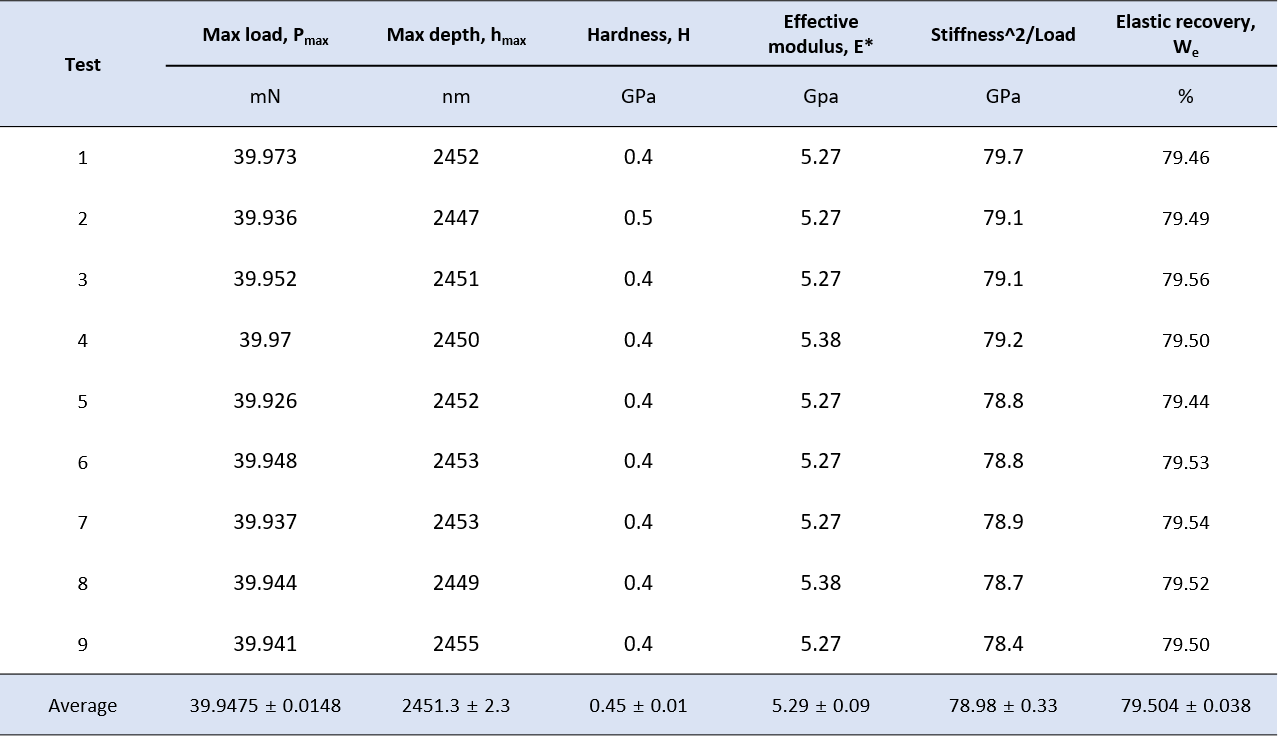


**Table S4.** Nano-indentation results for the CMSH.


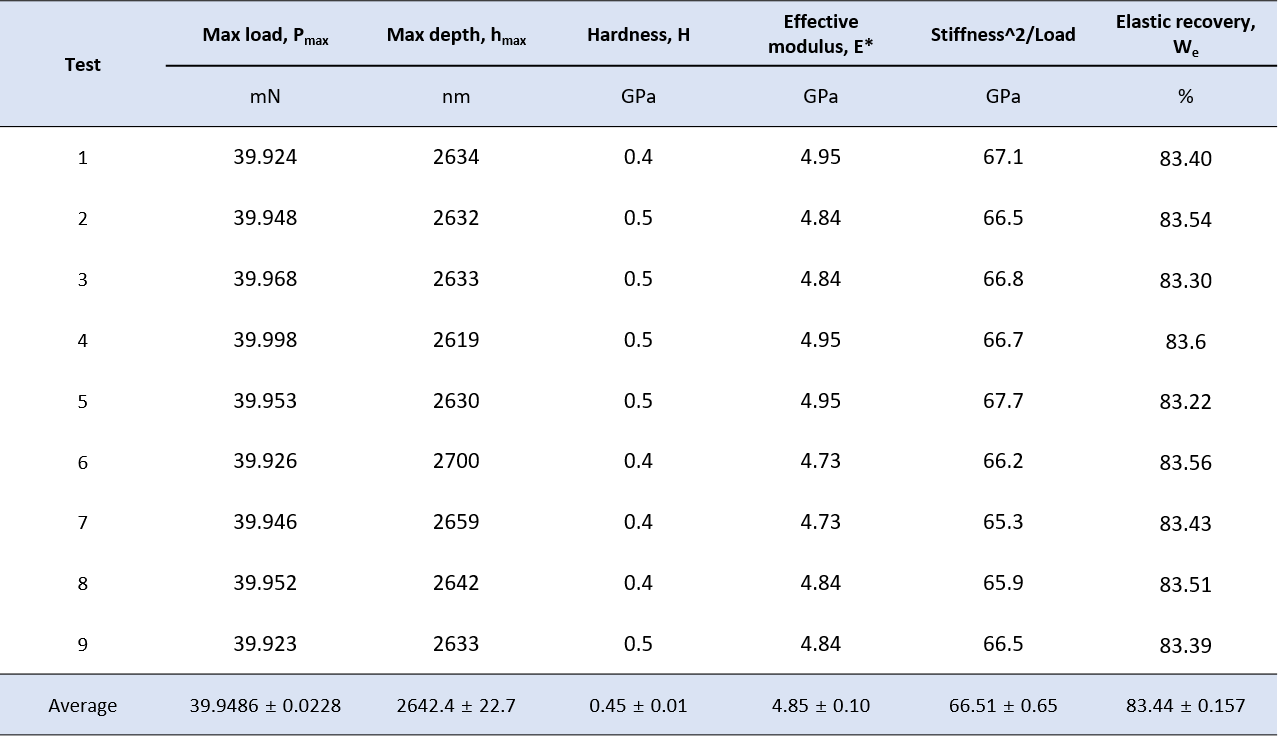

© 2017 by the authors. Submitted for possible open access publication under the
terms and conditions of the Creative Commons Attribution (CC BY) license (http://creativecommons.org/licenses/by/4.0/).
